# Defining ‘good health’

**DOI:** 10.18632/aging.101154

**Published:** 2016-12-29

**Authors:** Susan E. Erdman

**Affiliations:** Division of Comparative Medicine Massachusetts Institute of Technology, Cambridge, MA 02139, USA

**Keywords:** aging, oxytocin, microbiota, social health, spirituality

We all want to live a long life with ‘good health’. But what does that really mean? Clinicians often define ‘good health’ as the absence of disease. Indeed, modern biomedical research focuses on finding remedies for specific ailments, that, when absent, will yield ‘good health’.

Perhaps we should think about ‘good health’ in a different way. Essentially re-defining our health goal to include a more rewarding human experience; thus, living not only physically healthier lives, but also more meaningful and impactful lives. On the surface this objective sounds far-fetched, and impossible to define. And it would seem ridiculous to turn to microbes for a more meaningful life. Or would it?

Recent scientific data suggests a refined concept of ‘good health’ may be within our grasp. In fact, two recent studies by Buffington et al. (2016) [1] and Varian et al. (2016) [2] connect bacteria found in human breast milk with the molecule oxytocin, commonly referred to as the ‘love hormone’. Oxytocin is produced in the brain hypothalamus, and has roles in social bonding and reproduction. Emerging data suggest that combinations of microbes, oxytocin, and the immune system interact to dramatically impact overall health [2, 3]. Utilizing bacteria to up-regulate endogenous oxytocin overcomes therapeutic challenges of oxytocin's short half-life and obscure brain targets [4]. And the possibility of a safe and simple microbial strategy to overhaul health via oxytocin is too tantalizing to ignore. Indeed, oxytocin has been linked with well-being in many different ways including physical, mental, social, and spiritual health (Figure 1).

**Figure:**
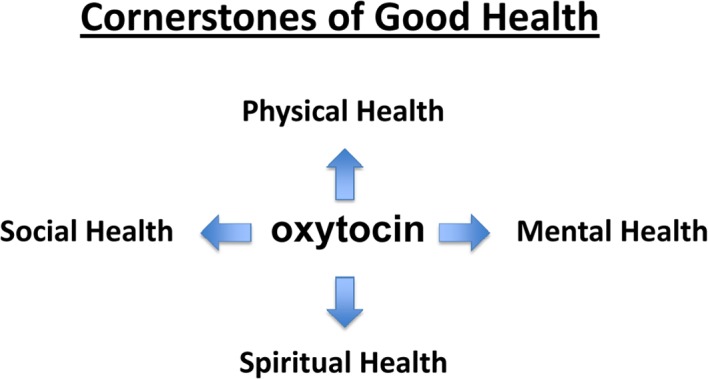


Examining this further, oxytocin benefits physical fitness in ways that are relatively easy to measure but too numerous to list here in their entirety. Among these are promoting robust musculature [5] with lower risk for obesity and diabetes [6], and improving immune function and wound repair capacity [3]. The resulting reinforcement of integument throughout the body protects against environmental challenges [3] yielding a more graceful aging process.

But there's more than just that. In terms of mental health, oxytocin is anxiolytic thus serving to reduce stress and convey a sense of enjoyment [7]. By stimulating reward centers of the brain, oxytocin biases the nervous system toward pleasure and a more optimistic attitude [1, 2]. Thus, oxytocin creates the perception of a more pleasant and rewarding life experience.

Oxytocin is also the hormone of good social health [1, 4], best recognized in terms of love and trust. This hormone promotes romantic bonds that build families, unifies mothers and their children, and builds friendships extending to patriotism in complex societies. In a nutshell, these are relationships that we would die for; relationships that motivate us toward serving the ‘greater good’ with sense of purpose and kindness toward others. Oxytocin gives life a social richness that money cannot buy.

In the spiritual realm, the hormone oxytocin is recognized for roles in meditation, appreciation of music, and organized religions [8]. These are among the most intangible effects of oxytocin, imparting an otherworldly sense of connectedness with the universe, and extending to visions of extraordinary insight and theoretical leaps throughout human history. The molecular basis of oxytocin may simply facilitate retrieval of ancestral (instinct) and early life memories for host survival advantage. Yet, this transcendental aspect of oxytocin raises the intriguing possibility of a deeply enriched human existence.

Taken together, these accumulated data motivate our revised definition of ‘good health’ to include a sense of purpose for a more meaningful life. It is simply amazing that exposure to a microbe once commonplace in humans boosts endogenous oxytocin levels and imparts a ‘microbial hug’ in animal models [4]. This microbe-host oxytocin symbiosis has yet to be proven in human subjects.

If our ultimate goal is a healthy and purposeful life, then it follows logically that our microbial passengers and oxytocin hold important keys in our quest.
